# Whole-Exome Sequencing Identifies Pathogenic Germline Variants in Patients with Lynch-Like Syndrome

**DOI:** 10.3390/cancers14174233

**Published:** 2022-08-31

**Authors:** Wellington dos Santos, Edilene Santos de Andrade, Felipe Antonio de Oliveira Garcia, Natália Campacci, Cristina da Silva Sábato, Matias Eliseo Melendez, Rui Manuel Reis, Henrique de Campos Reis Galvão, Edenir Inez Palmero

**Affiliations:** 1 Molecular Oncology Research Center, Barretos Cancer Hospital, Barretos 14784-400, São Paulo, Brazil; 2 Laboratory of Molecular Diagnosis, Barretos Cancer Hospital, Barretos 14784-400, São Paulo, Brazil; 3 Department of Molecular Carcinogenesis, National Cancer Institute, Rio de Janeiro 20231-050, Rio de Janeiro, Brazil; 4 Life and Health Sciences Research Institute (ICVS), Medical School, University of Minho, 4710-057 Braga, Portugal; 5 ICVS/3B’s-PT Government Associate Laboratory, 4710-057 Braga, Portugal; 6 Oncogenetics Department, Barretos Cancer Hospital, Barretos 14784-400, São Paulo, Brazil; 7 Department of Genetics, National Cancer Institute, Rio de Janeiro 20231-050, Rio de Janeiro, Brazil

**Keywords:** hereditary colorectal cancer, Lynch-like syndrome, cancer predisposition

## Abstract

**Simple Summary:**

A significant proportion of families with a clinical suggestion of Lynch syndrome and screened for the known MMR genes remain without a molecular diagnosis. These patients, who generally show a suggestive family pedigree or early-onset tumors with MMR deficiency and no detectable germline variants, are referred to as having Lynch-like syndrome. To investigate underlying and potentially predisposing variants related to Lynch-like syndrome, we performed whole-exome sequencing in patients with clinical criteria for Lynch syndrome, MMR deficiency and without germline variants. This approach allowed for the identification of new variants potentially associated with Lynch-like syndrome, providing new clues to explain the familial predisposition to Lynch syndrome-related tumors in these patients, which could lead to new screening strategies for the identification of families at risk of developing cancer.

**Abstract:**

Lynch syndrome (LS) is the most common hereditary colorectal cancer (CRC) syndrome, characterized by germline pathogenic variants in mismatch repair (MMR)-related genes that lead to microsatellite instability. Patients who meet the clinical criteria for LS and MMR deficiency and without any identified germline pathogenic variants are frequently considered to have Lynch-like syndrome (LLS). These patients have a higher risk of CRC and extracolonic tumors, and little is known about their underlying genetic causes. We investigated the germline spectrum of LLS patients through whole-exome sequencing (WES). A total of 20 unrelated patients with MMR deficiency who met the clinical criteria for LS and had no germline variant were subjected to germline WES. Variant classification was performed according to the American College of Medical Genetics and Genomics (ACMG) criteria. Pathogenic/likely pathogenic variants were identified in 35% of patients in known cancer genes such as *MUTYH* and *ATM*. Besides this, rare and potentially pathogenic variants were identified in the DNA repair gene *POLN* and other cancer-related genes such as *PPARG*, *CTC1*, *DCC* and *ALPK1*. Our study demonstrates the germline mutational status of LLS patients, a population at high risk of colorectal cancer.

## 1. Introduction

Hereditary cancer represents an important portion of the global cancer burden [1], but only a minority of such cases are attributed to known germline pathogenic variants and/or cancer-predisposing syndromes [2]. Lynch syndrome (LS) is the most common predisposing syndrome associated with colorectal cancer (CRC), accompanied by an increased risk of extracolonic cancers, such as endometrium, stomach, ovary, pancreas, ureter or renal pelvis, biliary tract, brain (mainly glioblastoma) and small bowel [3]. According to the currently accepted consensus, LS is characterized by germline variants in genes related to DNA mismatch repair (MMR), mainly the *MLH1*, *MSH2*, *MSH6* and *PMS2* genes, which lead to MMR deficiency and consequent tumors with microsatellite instability (MSI) [3]. Besides this, *EPCAM* deletions are also a known cause of Lynch syndrome [3].

LS patients generally present fulfilling the Amsterdam criteria or one of the revised Bethesda guidelines [3] and with a pathogenic germline variant in MMR genes. However, 30% of the families with a clinical suggestion of LS and screened for the common MMR genes remain without a molecular diagnosis [3]. This subset of patients, who generally show a suggestive family pedigree or early-onset tumors with MMR deficiency and no detectable germline mutation or hypermethylation in the MMR genes, are referred to as having Lynch-like syndrome (LLS) [4]. Although the clinicopathological features of LLS patients appear to differ from those of LS patients [5] and resemble those of patients with sporadic tumors [6], the risk of colorectal cancer in these patients and their families is reported to be higher than that of sporadic tumors [7]. Furthermore, patients with LLS are often diagnosed at a younger age than patients with sporadic tumors [6,8]. This indicates, at least in part, a hereditary component of LLS.

A previous study from our group [9] evaluated 323 probands with a family history suggestive of LS. Among those, 134 tumors were MMR-deficient. Genetic testing was performed on 127 of them, and 65 (51%) did not have a pathogenic alteration at the *MLH1*, *MSH2*, *MSH6*, *PMS2*, or *EPCAM* gene, even though their tumors had MSI and loss of expression of either MMR protein, as indicated by IHC.

The underlying germline mutation spectrum of LLS is poorly explored. Some studies reported the presence of biallelic germline variants in the *MUTYH* gene in LLS cases [10,11], and *MUTYH*-associated polyposis can overlap with the LS phenotype by somatic inactivation of MMR genes [10]. Beyond this, LLS patients carrying *POLE* and *POLD1* germline variants have also been identified [12,13]. The presence of germline variants in DNA repair genes, such as *MCM8*, *MCM9*, *WRN*, *MCPH1*, *BARD1*, *REV3L*, *EXO1*, *POLD1*, *RFC1*, *RPA1* and *MLH3*, has additionally been reported in patients with LLS [8,13,14].

In that context, we performed whole-exome sequencing (WES) in patients with an MMR deficiency without germline variants and identified new variants possibly associated with LLS development.

## 2. Materials and Methods

### 2.1. Patient Selection

**Figure 1:**
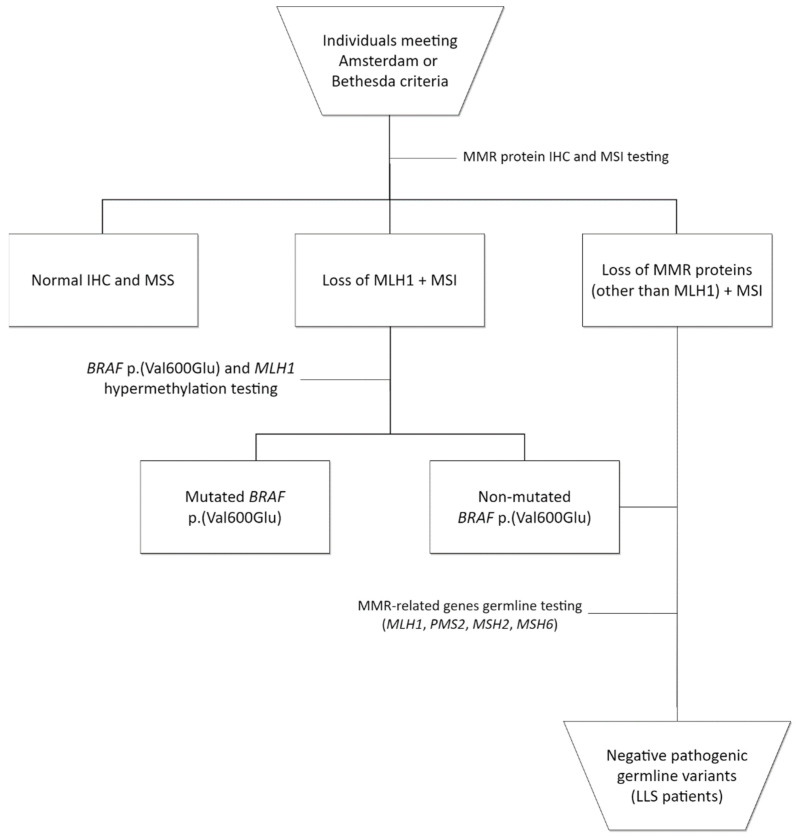
Flowchart of Lynch-like syndrome (LLS) patients’ identification and inclusion. IHC: immunohistochemistry; MSI: microsatellite instability.

Twenty patients identified at the Oncogenetics Department of Barretos Cancer Hospital were included in this study [15]. Patients were included after signing an informed consent form, and the study was approved by the Barretos Cancer Hospital Institutional Review Board (protocol CAAE: 56164716.9.0000.5437). The patient selection followed the Lynch syndrome strategy as previously reported [9]. Briefly, samples from patients meeting the Amsterdam or Bethesda criteria underwent immunohistochemistry (IHC) for the four MMR-related proteins (MLH1, PMS2, MSH2 and MSH6) and microsatellite instability (MSI) analysis. Patients with MMR-deficient tumors for PMS2, MSH2 or MSH6 underwent germline genetic testing for the respective gene. Meanwhile, patients with MLH1-deficient tumors were subjected to germline genetic testing only if they had a wild-type result in *BRAF* p. (Val600Glu) (*BRAF* V600E) analysis, regardless of their *MLH1* hypermethylation status. Patients with an absence of germline variants in any of the MMR-related genes and with loss of MMR protein expression were included in this study (Figure 1).

### 2.2. DNA Isolation and Whole-Exome Sequencing

Genomic DNA was isolated from peripheral blood using the QIAmp DNA Blood Mini Kit for the QIAcub automated platform (QIAGEN, Hilde, Germany) following the manufacturer’s instructions. The DNA quantity and quality were assessed by a Qubit^®^ 2.0 Fluorometer (Thermo Fisher Scientific, Waltham, MA, USA). WES was conducted by SOPHiA™ genetics using an Illumina NovaSeq sequencer (Illumina, San Diego, CA, USA) with a Whole Exome Solution Kit (version 1), including 203,058 target regions and 40,907,213 bp in 19,682 genes. The mean coverage of sequencing was 150× (99.6% above 10×, 99.4% above 20×, 99.3% above 20 and 30× and 99.3% above 40× and 50×).

### 2.3. Sequence Quality Control, Alignment and Variant Calling

Determination of the quality of reads, alignment and variant calling were performed as previously described [16]. The quality of reads was accessed by FASTQC [17], trimmed by Cutadapt [18] and mapped against the human genome reference (build GRCh37/hg19) using the Burrows-Wheeler Aligner (BWA, version 0.7.17) [19]. Postprocessing alignment was performed using Picard [20] for read duplication removal, and the Genome Analysis Toolkit (GATK) [21] was used for quality score recalibration. Variant calling was performed by the HaplotypeCaller [22].

### 2.4. Variant Annotation and Classification

**Figure 2:**
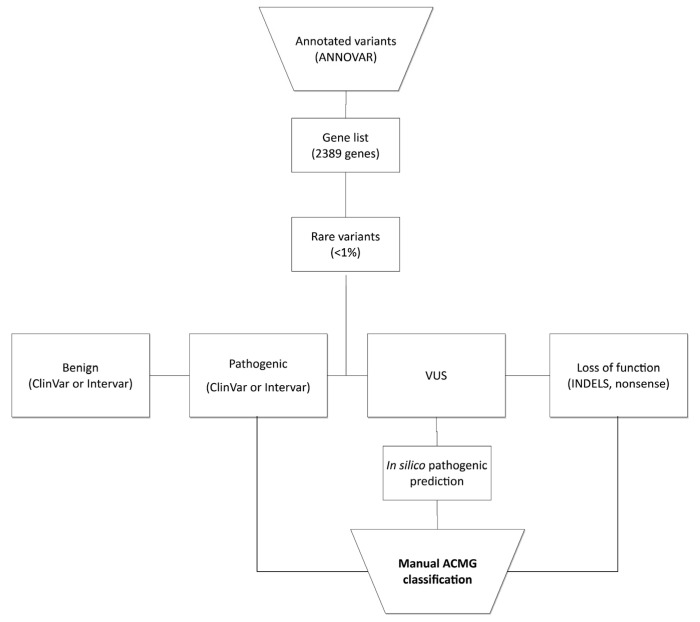
Flowchart of decision process for variant prioritization.

Variant annotation was performed by ANOVA. We analyzed a selected set of 2389 genes [16] (Supplementary Table S1), built from cancer-related genes (COSMIC [23], UniProt [24] and DISEASES databases [25]), hereditary syndrome cancer-related genes (extracted from commercial panels, GeneCards [26] and the Genetics Home Reference database [27]) and DNA repair genes (from Das and colleagues’ study [28]). We developed an analytical pipeline to filter variants for manual classification (Figure 2). Briefly, variants were filtered to remove those with fewer than 30 reads and a variant allele frequency below 25%. Populational databases (ABraOM and gnomAD) were used to remove variants with a minor allele frequency >1%. Pathogenic variants (defined by ClinVar or Intervar), loss-of-function variants (indels and nonsense variants) and variants of uncertain significance (VUS) with an in silico pathogenic score (REVEL >0.7 or M-CAP >0.025 for missense variants and Human Splicing Finder (HSF) for splicing variants) were selected for manual classification. Two independent researchers (the first and last authors of this study) manually classified the selected variants as benign or likely benign (I or II), VUS (III), likely pathogenic or pathogenic (IV or V) following ACMG criteria [29]. All selected variants were subjected to visual exploration in the Integrative Genomics Viewer (IGV) [30]. Variants classified as IV or V were confirmed by bidirectional Sanger sequencing.

### 2.5. Statistical Analysis

Statistical analyses were performed using SPSS (v. 23) and R (v. 3.6.1) software. Descriptive data were expressed by a number, percentage, mean and standard deviation. Age comparisons between groups were performed by analysis of variance (ANOVA). Numbers of tumors were compared using the Kruskal–Wallis test. The chi-squared or Fisher’s exact test was performed to compare potentially pathogenic/likely pathogenic variants and tumor features.

## 3. Results

### 3.1. Patients

We included in our study a total of 20 patients with MMR deficiency but without pathogenic germline variants in the MMR-associated genes (Table 1). Most patients were female (60%), and the mean age of the first diagnosed tumor was 48 years (SD = 7.7). CRC was the first diagnosed tumor in 75% of patients (*n* = 15), and otherwise, the extracolonic tumors first diagnosed were endometrial (*n* = 2), ovarian (*n* = 2) and gastric (*n* = 1). Five patients were diagnosed with a second tumor; those included CRC, endometrium, breast and non-melanoma skin (Table 1). Amsterdam clinical criteria were fulfilled by 25% of patients. A total of 90% of patients had a family history of cancer, and 75% had LS-related tumors in the family.

### 3.2. Germline Variants’ Profile

**Figure 3:**
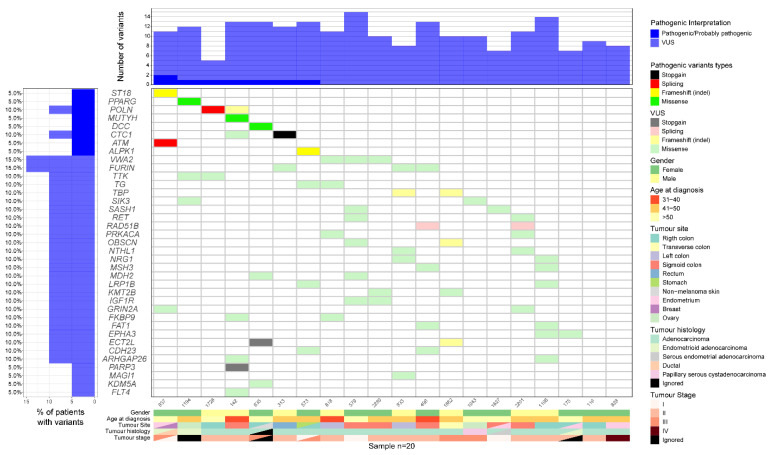
Representative plot of patients carrying pathogenic and likely pathogenic variants (genes on the top) and VUS (only for genes with over 5% VUS in our patients with Lynch-like syndrome).

After filtering out variants, we found a total of 319 germline variants for manual prioritization on 2389 analyzed genes. Manual classification using ACMG criteria resulted in 33.5% of variants being classified as benign or likely benign (107/319), 63.9% variants of uncertain significance (204/319, Supplementary Table S2) and 2.5% pathogenic or likely pathogenic variants (8/319). Pathogenic/likely pathogenic variants were present in 35% of patients (7/20, Figure 3 and Table 2). These patients with pathogenic variants did not differ from patients without pathogenic variants concerning the age at first diagnosed tumor (mean age of 48.3 vs. 48.2, *p* = 0.974), number of family tumors (mean number of 3.7 vs. 6.7 tumors, *p* = 0.193), tumor grade (*p* = 0.650) or tumor stage (*p* = 0.854).

### 3.3. Germline Variants’ Classification

Variants classified as pathogenic and likely pathogenic are shown in Table 2. A heterozygous pathogenic missense variant in the *MUTYH* gene (NM_001128425.2:c.1187G > A p.(Gly396Asp), Table 2) was found in a patient with CRC who was diagnosed at age 39 (ID 142, Table 2) and had a familial history of CRC of paternal lineage and esophageal cancer of maternal lineage (Supplementary Table S2 and Figure S1). The patient’s tumor showed loss of MLH1/PMS2 expression and isolated loss of MSH6 expression. This patient also showed a heterozygous nonsense variant at the *PARP3* gene that was classified as VUS, as well as 11 additional variants classified as VUS (Supplementary Table S3).

A heterozygous pathogenic splicing variant was found on a DNA polymerase type-A family member, *POLN* (NC_000004.11(NM_181808.2):c.1375-2A > G) on patient ID 1728 (Table 2). In addition, a VUS in another DNA repair gene (*ERCC5*), as well as variants on the *E2F7*, *GRHL2* and *TTK* genes (Supplementary Table S3), were identified in the same patient. This patient had CRC diagnosed at age 57, with loss of PMS2 expression and weak MLH1 expression, but did not show a history of tumors in the family (Supplementary Table S2, Supplementary Figure S2).

A *CTC1* heterozygous pathogenic nonsense variant (NM_025099.6:c.19C > T p. (Gln7Ter), Table 2) was found in a patient (ID 313) who had CRC with isolated loss of MSH6, as was diagnosed at age 48; the patient had no LS-related tumors in the family (Supplementary Table S2, Supplementary Figure S3). Yet, this patient also showed a VUS with a high score for pathogenic prediction in the *RBL1* gene and a truncating VUS in the *PCM1* gene, which are both cancer-related genes (Supplementary Table S3). Further to this, we classified nine variants as VUS in several other cancer-related genes (Supplementary Table S3).

The *DCC* gene showed a heterozygous likely pathogenic missense variant possibly affecting the splice site at the end of exon 11 (NM_005215.4:c.1861G > A p.(Val621Met), Table 2) that we classified as likely pathogenic. The patient carrying this variant (ID 635) had CRC with loss of PMS2/MSH6 expression at age 50 and a nonmelanoma skin tumor diagnosed at age 56 (Table 2 and Supplementary Figure S4). The family of the patient did not show a history of tumors (Supplementary Table S2, Supplementary Figure S4). In addition to the likely pathogenic variant on the *DCC* gene, we also identified a truncating VUS in the *ECT2L* gene, along with 11 variants classified as VUS in cancer-related genes (Supplementary Table S3).

### 3.4. Variants in Patients with Loss of MLH1 or PMS2

We identified 10 patients with tumors not expressing MLH1 or PMS2, six of whom had hypermethylation in the *MLH1* promoter region. With one inconclusive exception, all *MLH1*-methylated cases were *BRAF* p. (Val600Glu) wild-type. We did not find any difference between *MLH1*-methylated cases and *MLH1*-nonmethylated cases with an MLH1 or PMS2 expression deficiency regarding the mean age at first diagnosis (mean age of 48 vs. 55.1, *p* = 0.591), number of family tumors (mean number of 4.8 vs. 8 tumors, *p* = 0.118) or presence of potentially pathogenic variants (50% vs. 25%, *p* = 0.571).

Potentially pathogenic variants were identified in three *MLH1*-hypermethylation cases. A likely pathogenic missense variant at the *PPARG* gene (NM_015869.5:c.1230C > A p.(Ser410Arg), Table 2) was found in a patient (ID 1194) with an ovarian tumor diagnosed at age 44. This tumor showed MSI without loss of MMR proteins and methylation of the *MLH1* gene. The mother of the patient was diagnosed with meningioma at age 71, and the paternal grandfather had a gastric tumor (Supplementary Table S2 and Figure S5). We also identified 11 VUS in this patient, including a missense variant on the *FANCA* gene, which is involved in DNA repair damage (Supplementary Table S3).

Another likely pathogenic frameshift variant was identified in the *ALPK1* gene (NM_001102406.2:c.3428_3431del p. (Asn1143ThrfsTer5), Table 2). This variant was identified in a patient (ID 573) with a gastric tumor diagnosed at age 44 and CRC diagnosed at age 49 with loss of MLH1/PMS2 expression and methylated *MLH1*. The patient’s brother had a pharynx tumor, and her paternal lineage developed tumors of the breast (paternal aunt) and stomach (paternal grandmother, Supplementary Table S2 and Figure S6). We also identified 12 VUS in this patient, including a missense mutation with a high score of pathogenic prediction in the *EPHA5* gene and three truncating VUS in the *NBPF3*, *NINL* and *RETN* genes (Supplementary Table S3).

Finally, the other patient with an *MLH1* methylated tumor in which pathogenic variants were identified was ID 837. In this patient, with an endometrial tumor diagnosed at age 53 and loss of MLH1/PMS2 expression, we identified two variants classified as pathogenic and likely pathogenic in the *ATM* and *ST18* genes, respectively. This patient was further diagnosed with breast cancer at age 58. The *ATM* gene had a splicing variant (NC_000011.9(NM_000051.3):c.3993 + 1G > A) classified as pathogenic, and the ST18 gene had a frameshift variant (NM_014682.2:c.2093del p. (Lys698SerfsTer24), Table 2) classified as likely pathogenic (Table 2). In addition, this patient harbored 9 VUS (Supplementary Table S3), including two missense variants with high scores of pathogenic predictions in the *RPN1* and *TMC8* genes, respectively. The patient’s sister had a breast tumor at age 56, and her maternal lineage showed tumors of the throat, prostate and intestine (Supplementary Table S2 and Figure S7).

In addition to the three *MLH1*-methylated cases harboring potentially pathogenic germline variants, we also had three *MLH1*-methylated cases without. A patient with CRC with loss of MLH1/PMS2 expression diagnosed at age 41 (ID 1196) and a family history of pancreatic tumor (father, Supplementary Table S2) was identified as carrying VUS in the MMR-related *MSH3* gene (NM_002439.5:c.1777C > T p. (Arg593Trp) and a *CHEK2* VUS (NM_007194.4:c.1427C > T p. (Thr476Met), Supplementary Table S3). We also identified a high score of pathogenic prediction for missense variants in the *EPHA3*, *LRP1B* and *RAB5A* genes in this patient (Supplementary Table S3). Another *MLH1*-methylated case was identified in a patient with an ovarian tumor at age 56 and CRC with loss of MLH1/PMS2 expression at age 60 (ID 1043), and she had a family history of LS-related tumors (Supplementary Table S2). This patient was identified to have a missense VUS with a high score of pathogenic prediction in the *ERCC6L2* and *SIK3* cancer-related genes in addition to VUS in the *SMAD6* CRC-related gene (Supplementary Table S3). A third *MLH1*-methylated case carrying only VUS was observed in a patient (ID 828, Supplementary Table S3) diagnosed at age 46 with an endometrial tumor, with loss of MLH1/PMS2 expression and a family history of endometrial, prostate and unidentified tumors (mother, father and paternal uncle, respectively, Supplementary Table S2).

### 3.5. Variants of Uncertain Significance (VUS)

VUS were found in several key genes (Supplementary Table S3), with 43 genes harboring either a high score of pathogenic prediction (REVEL score > 0.7) or truncation variants. In addition to the VUS in the MMR-related *MSH3* gene on patient ID 1196, we found a high pathogenic score prediction or truncation VUS on DNA repair genes *CCNH* (ID 116), *TP53BP1* (ID 175), *RAD51B* (IDs 2201 and 496), *NTHL1* (IDs 2201 and 933), *POLH* (ID 496) and *RAD54L* (ID 579, Supplementary Table S3).

## 4. Discussion

In the present study, we performed WES on germline DNA from patients with MSI positivity and loss of MMR protein expression but without germline MMR pathogenic variants. To the best of our knowledge, this is the first study to explore germline identification through WES for LLS patients in a Brazilian population. WES technologies have become accessible and have been integrated into clinical practice in recent years [31]. Although this approach has several challenges, such as data management, incidental findings and variant prioritization and/or interpretation [32], WES can be useful to uncover the underlying genetic basis of cancer predisposition [33,34].

Through our WES approach, we identified 35% of LLS patients harboring potentially pathogenic variants in cancer-related, hereditary or DNA repair genes. Previous studies that investigated the germline basis for LLS identified a wide range of variants with the potential for cancer predisposition [10,14,35]. Using WES, Xicola and colleagues identified a similar frequency of potentially pathogenic variants in DNA repair-related genes (36.4%) to that found in the current study [8]. Other potentially pathogenic germline variants have been linked to LLS patients, such as variants in *POLE* [35], MCM8 [14] and *MUTYH* [10,36].

An inherited biallelic mutation at the *MUTYH* gene is related to MUTYH-associated polyposis [37], and the missense *MUTYH* p. (Gly396Asp) variant that we found is related to abnormal MUTYH protein activity [38]. *MUTYH* monoallelic variant carriers had an approximately two-fold increased risk of colorectal cancer [39] and showed an increased risk of gastric, liver and endometrial tumors (3.34, 3.09 and 2.33, respectively) [40]. The prevalence of *MUTYH* monoallelic variants in LLS has previously been reported as 3.6% in LLS patients [10], similar to the frequency observed in our study. Furthermore, screening for *MUTYH* variants has been proposed for patients with MMR deficiency and the absence of MMR-related germline variants [10].

The polymerase *POLN* gene is involved in DNA cross-link repair and homologous recombination [41]. The variant present in our cohort is supposed to affect the splicing of *POLN* exon 12. The frequency of *POLN*-inactivating variants shown as increased in patients with pancreatic tumors compared to controls [42] and has a 6.9-fold increased risk of prostate tumors in the Chinese population [43]. An inactivating variant of the *POLN* gene has also been found in ovarian cancer patients, although the frequency did not differ significantly from controls [44]. Another variant affecting splicing sites was found in the *DCC* gene, which encodes a transmembrane protein involved in axonal guidance of neuronal growth and is frequently deleted or downregulated in CRC [45].

The *CTC1* gene encodes a component of the CST complex that plays a role in telomeric integrity [46]. Variants in the *CTC1* gene are associated with Coats plus syndrome [47], as well as cerebroretinal microangiopathy with calcifications and cysts [46]. Heterozygous deleterious germline variants at the *CTC1* gene have been found in myelodysplastic syndrome [48], and the nonsense mutation that we found here has been found in a patient with acute myeloid leukemia [49].

Several studies do not include *MLH1*-methylated cases in LLS germline investigations [8,14]. Yet, the presence of tumors with *MLH1* methylation does not exclude the presence of germline variants in LS patients [9]; we identified the presence of pathogenic variants in 50% of our *MLH1*-methylated cases. Interestingly, there were two pathogenic variants not previously reported in the literature, which were identified in *MLH1*-methylated cases from our study. These were a frameshift variant on *ALPK1*, a gene with downregulated expression in lung and colorectal tumors [50], and a frameshift variant on *ST18*, a gene with tumor-suppressing activity in breast tumors [51]. Another pathogenic variant identified in *MLH1*-methylated cases was a missense variant in the *PPARG* gene, which is a member of the peroxisome proliferator-activated receptor subfamily, missense variants of which have been found in a family with dyslipidemia and colonic polyp formation [52] and patients with endometrial carcinoma [53]. Additionally, another pathogenic variant identified in *MLH1*-methylated cases was a splicing *ATM* variant shown in patients with ataxia-telangiectasia [54], breast [55] and pancreatic [56] tumors.

Despite the interesting and novel findings, our work has certain limitations. The restricted analysis of a prebuilt gene set limited our work, meaning we could not engage in variant discovery outside this subset of genes. Additionally, the intrinsic restriction of WES technology meant we could not investigate intronic variants or regulatory regions outside exon sequences. Nor could we investigate tumor tissue mutations beyond the *BRAF* p. (Val600Glu) status, which would have provided further information on the loss of heterozygosity and pathogenicity evidence, as well as the possibility of MMR biallelic mutations. Finally, the small number of patients evaluated impacted the statistical significance of the germline findings and clinical associations. Yet, despite these limitations, this study makes an important contribution to the field, given that the Brazilian population is relatively understudied. Besides this, we identified promising candidate genes involved in DNA repair, apoptosis and metabolism, among other pathways, thus providing novel information on potential LLS-related pathways and an excellent premise for future studies and the discovery/validation of novel associations between genes and diseases.

## 5. Conclusions

To the best of our knowledge, this is the first study to investigate the germline basis for Lynch-like syndrome in Brazilian patients through WES. We reported the presence of potentially pathogenic variants that could explain the familial predisposition to Lynch syndrome-related tumors without a germline basis of MMR deficiency, including cases with *MLH1* methylation, which could support new screening strategies for the identification of families at risk of developing cancer.

## Figures and Tables

**Table 1:** Characteristics of patients with Lynch-like syndrome (LLS).

Characteristics (*n* = 20)	*n*	(%)
Gender		
Female	12	(60)
Male	8	(40)
Survival status		
Followed-up	19	(95)
Deceased	1	(5)
Mean age of first diagnosed tumor (SD)	48.2 (7.7)	
First diagnosed tumor		
Colorectal cancer	15	(75)
Endometrium	2	(10)
Ovary	2	(10)
Stomach	1	(5)
Mean age of second diagnosed tumor (SD)	59.2 (7)	
Second diagnosed tumor		
Colorectal cancer	1	(5)
Endometrium	2	(10)
Breast	1	(5)
Non-melanoma skin	1	(5)
Clinical criteria		
Amsterdam	5	(25)
Bethesda	8	(40)
Revised Bethesda	7	(35)

SD: standard deviation.

**Table 2:** Pathogenic (V) or likely pathogenic (IV) variants identified in our patients with Lynch-like syndrome.

ID Case	Gene	Pathogenic Variant	REVEL	AF	(Class) ^1^	Tumor Site	Age ^2^	Criteria Fulfilled
1194	*PPARG*	NM_015869.5:c.1230C > A p.(Ser410Arg)	0.767	1.60 × 10^−5^	(IV)	ovary	44	Bethesda *
142	*MUTYH*	NM_001128425.2:c.1187G > A p. (Gly396Asp)	0.954	3.00 × 10^−3^	(V)	colorectal	39	Bethesda
1728	*POLN*	NC_000004.11(NM_181808.2):c.1375-2A > G splicing variant	-	4.07 × 10^−6^	(V)	colorectal	57	Bethesda *
313	*CTC1*	NM_025099.6:c.19C > T p. (Gln7Ter)	-	1.68 × 10^−5^	(V)	colorectal	48	Bethesda *
573	*ALPK1*	NM_001102406.2:c.3428_3431del p. (Asn1143ThrfsTer5)	-	-	(IV)	stomach colorectal	44 49	Bethesda
635	*DCC*	NM_005215.4:c.1861G > A p. (Val621Met)	0.303	2.00 × 10^−4^	(IV)	colorectal non-melanoma skin	50 56	Bethesda *
837	*ATM*	NC_000011.9(NM_000051.3):c.3993 + 1G > A splicing variant	-	1.60 × 10^−5^	(V)	endometrium breast	53 58	Bethesda *
	*ST18*	NM_014682.2:c.2093del p. (Lys698SerfsTer24)	-	-	(IV)			

Af: allele frequency on gnomAD; ACMG: American College of Medical Genetics and Genomics criteria; ^1^ Variant classification according to ACMG criteria; ^2^ Age at first diagnosed tumor; * Revised Bethesda Guidelines.

## Data Availability

Variants classified will be deposited in the ClinVar database. Data that support the findings are not readily available due to privacy and ethical restrictions. Requests for data access should be directed to the corresponding author, Edenir Inez Palmero.

## Supplementary Materials

The following supporting information can be downloaded at: https://www.mdpi.com/article/10.3390/cancers14174233/s1, Figures S1–S7: Representative pedigrees of the families of patients carrying putative pathogenic or likely pathogenic variants; Table S1: List of genes analyzed in the present study; Table S2: Family history and pathogenic (V) or likely pathogenic (IV) variants identified in our patients with Lynch-like syndrome; Table S3: List of VUS classified by ACMG manual curation in our patients with Lynch-like syndrome.
