# Supplementary material for: Whole-Exome Sequencing Identifies Pathogenic Germline Variants in Patients with Lynch-Like Syndrome
Source: Cancers (Basel). 2022 Aug 31;14(17):4233. doi: 10.3390/cancers14174233 (PMC9454535; doi:10.3390/cancers14174233)
Supplement: Supplementary file 1 [file cancers-14-04233-s001.zip › cancers-1703647-Supplementary Figures.pdf]

Whole-exome sequencing identifies pathogenic germline variants in patients with Lynch-like syndrome

Wellington dos Santos [1], Edilene Santos de Andrade [1], Felipe Antonio Oliveira Garcia [1], Natália Campacci [1], Cristina da Silva Sábato [2], Matias Eliseo Melendez [1,3], Rui Manuel Reis [1,4,5], Henrique de Campos Reis Galvão[6] and Edenir Inez Palmero [1,7,*]

[1] Molecular Oncology Research Center, Barretos Cancer Hospital, Barretos, São Paulo, Brazil
[2] Laboratory of Molecular Diagnosis, Barretos Cancer Hospital, Barretos, São Paulo, Brazil
[3] National Cancer Institute; Rio de Janeiro – RJ, Brazil
[4] Life and Health Sciences Research Institute (ICVS), Medical School, University of Minho, Braga, Portugal
[5] ICVS/3B's-PT Government Associate Laboratory, Braga/Guimarães, Portugal
[6] Oncogenetics Departament, Barretos Cancer Hospital, Barretos, São Paulo, Brazil
[7] Department of Genetics, Brazilian National Cancer Institute, Rio de Janeiro, Brazil
* Correspondence: edenirip@yahoo.com.br; Tel.: +55-17-3321-6600 (ext 7057)

This file contains the representative pedigrees of the families of patients carrying putative pathogenic or likely pathogenic variants.

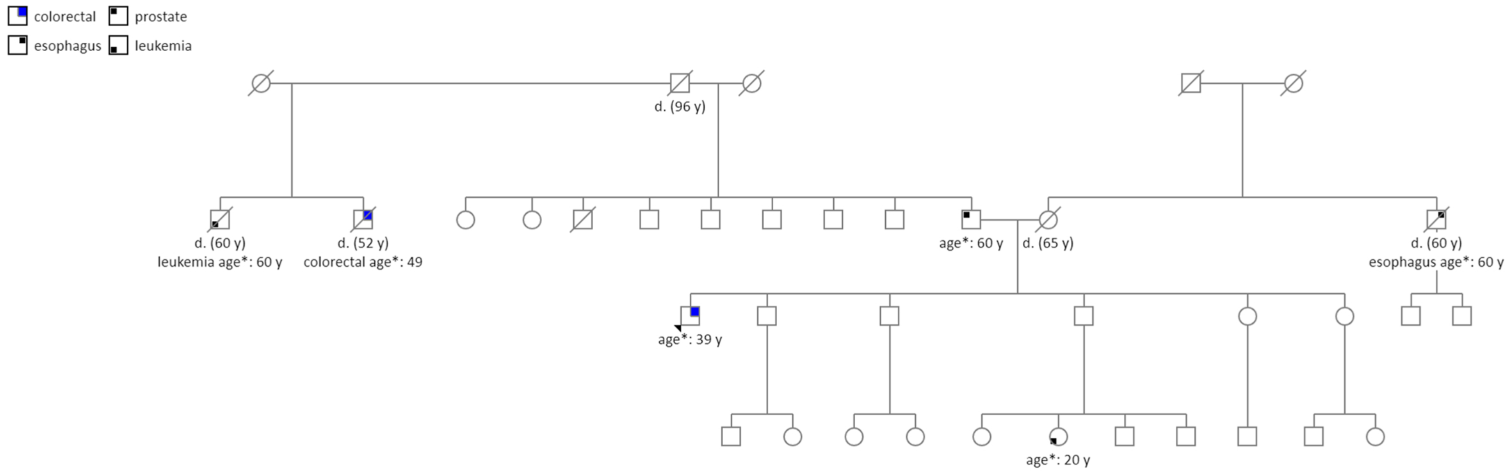

Supplementary Figure S1. Representative pedigree of the family with the patient ID 142, that carry a heterozygous pathogenic missense variant on *MUTYH* gene; d.: age of death; *: age of diagnosed tumour.

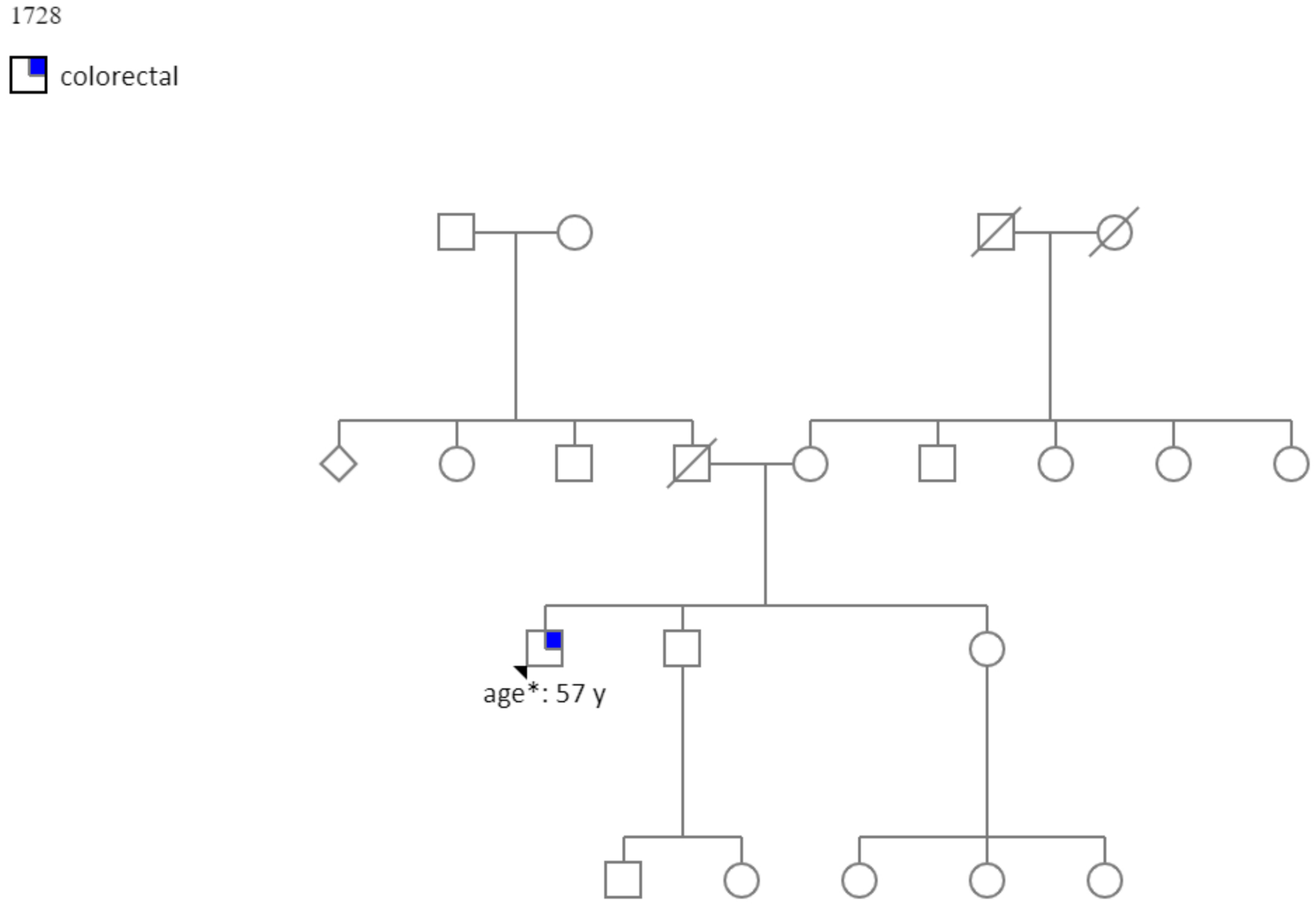

Supplementary Figure S2. Representative pedigree of the family with the patient ID 1728, that carry a heterozygous pathogenic splicing variant on *POLN* gene ; d.: age of death; *: age of diagnosed tumour.

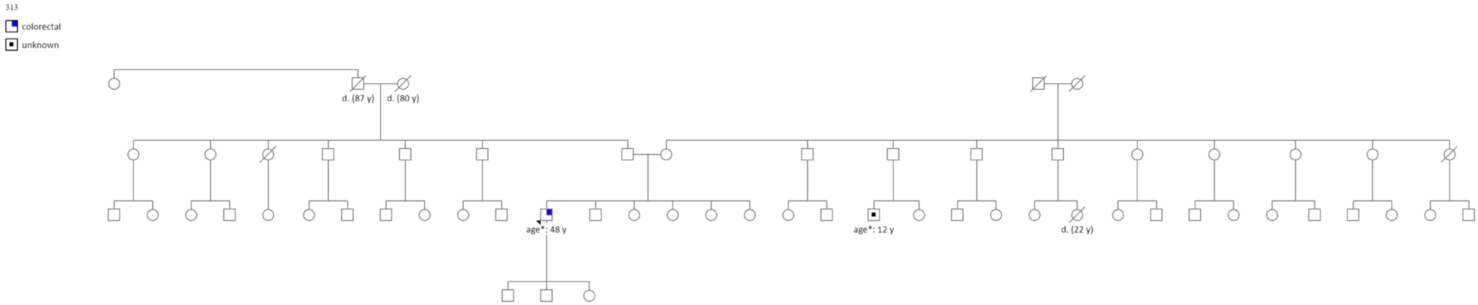

Supplementary Figure S3. Representative pedigree of the family with the patient ID 313, that carry a heterozygous pathogenic nonsense variant on *CTC1* gene; d.: age of death; *: age of diagnosed tumour.

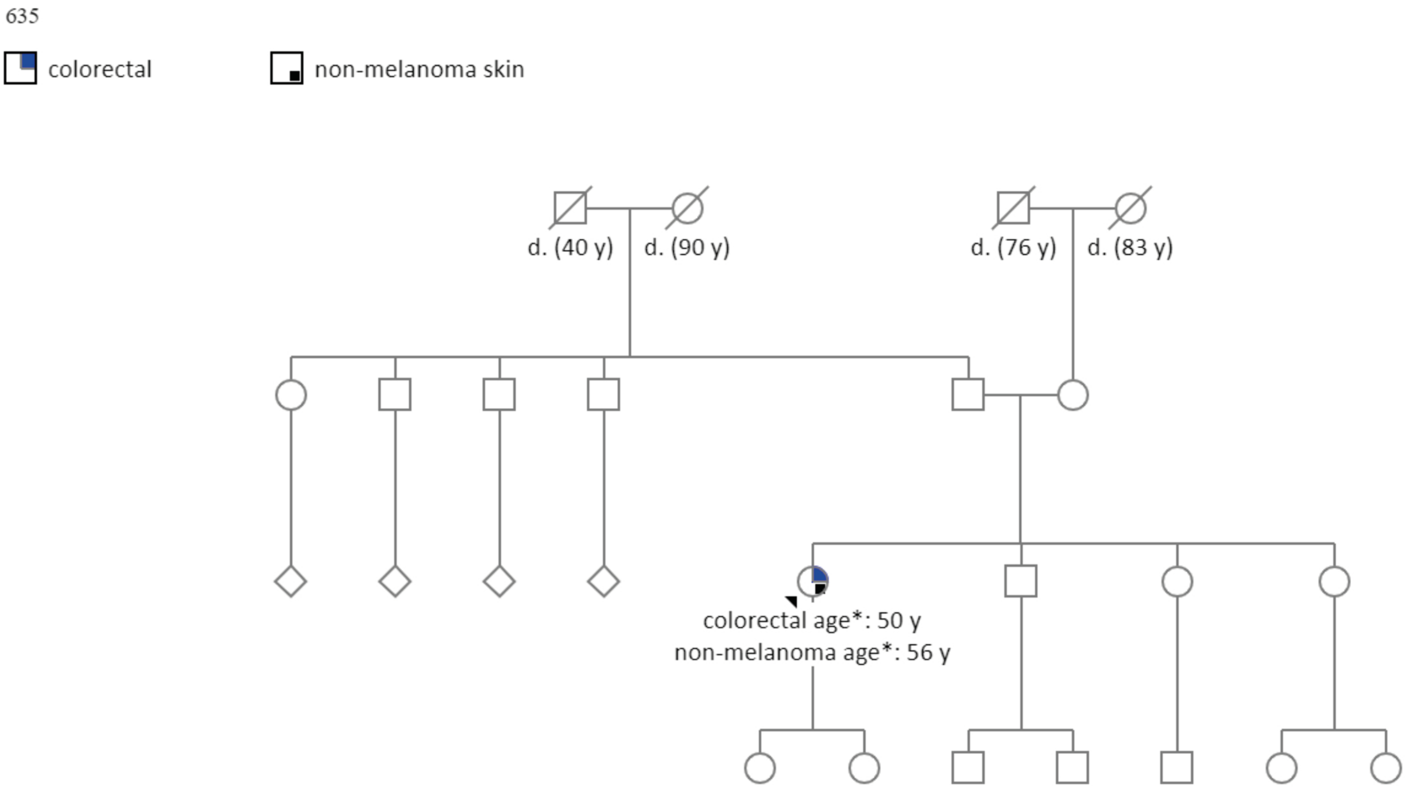

Supplementary Figure S4. Representative pedigree of the family with the patient ID 635, that carry a heterozygous llikely pathogenic missense variant on *DCC* gene; d.: age of death; *: age of diagnosed tumour.

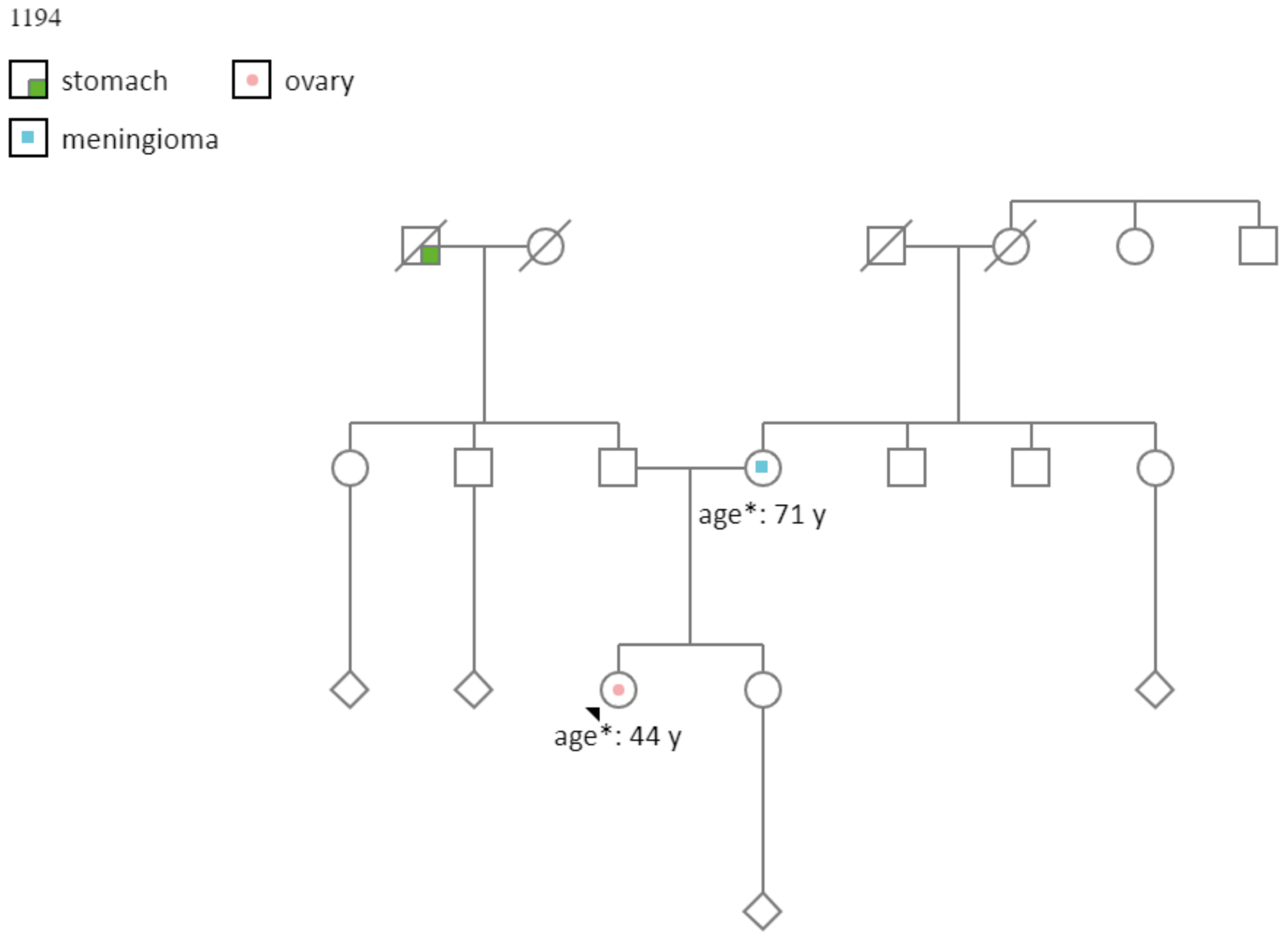

Supplementary Figure S5. Representative pedigree of the family with the patient ID 1194, that carry a heterozygous likely pathogenic missense variant on *PPARG* gene; d.: age of death; *: age of diagnosed tumour.

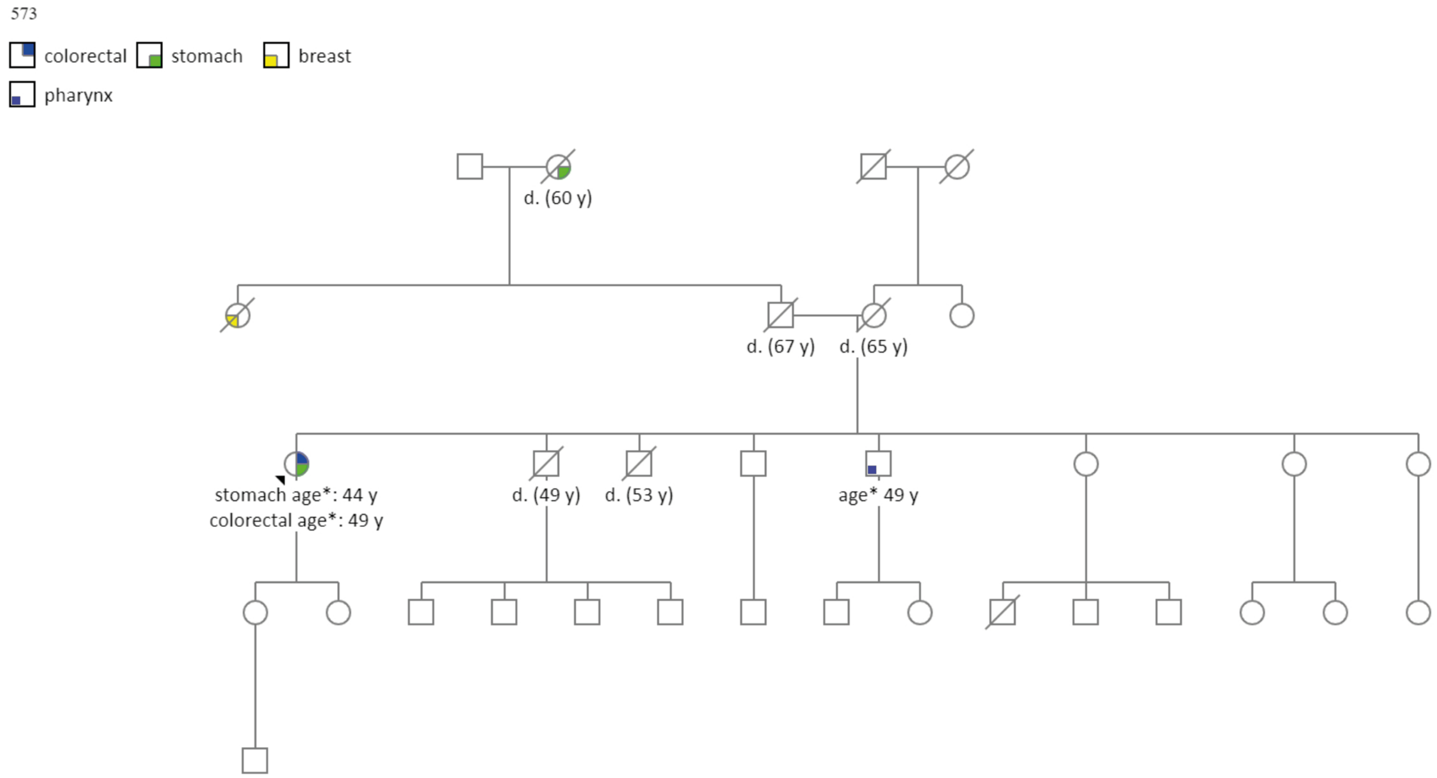

Supplementary Figure S6. Representative pedigree of the family with the patient ID 573, that carry a heterozygous likely pathogenic frameshift variant on *ALPK1* gene; d.: age of death; *: age of diagnosed tumour.

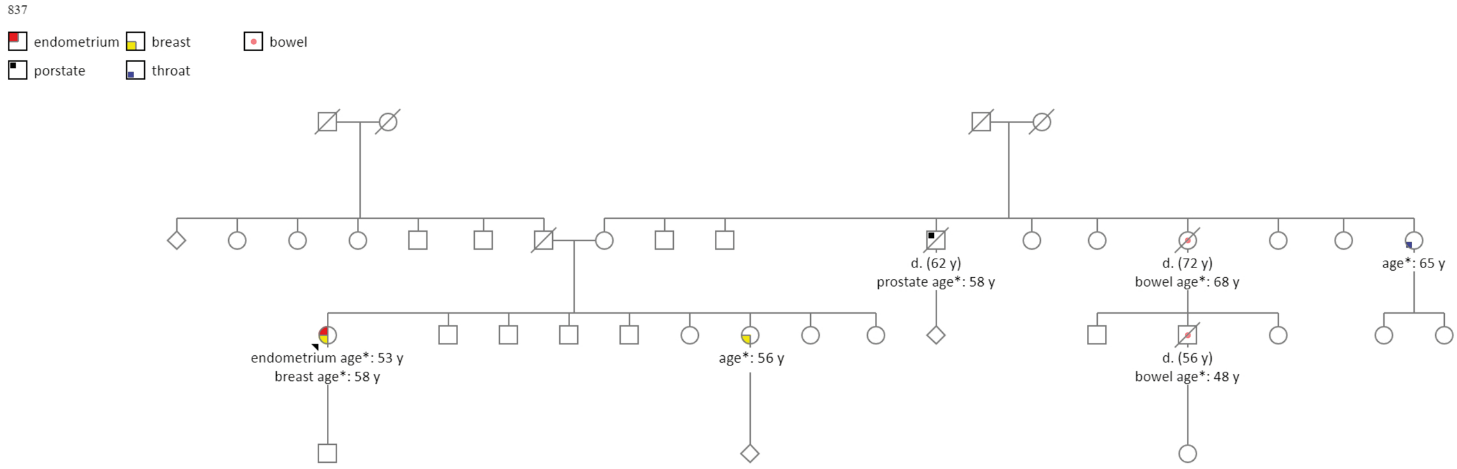

Supplementary Figure S7. Representative pedigree of the family with the patient ID 837, that carry a heterozygous pathogenic splicing variant on *ATM* gene and a heterozygous likely pathogenic frameshift variant on *ST18* gene; d.: age of death; *: age of diagnosed tumour.
